# Single-position prone lateral transpsoas interbody fusion with Mazor robotic guidance

**DOI:** 10.3171/2025.4.FOCVID24148

**Published:** 2025-07-01

**Authors:** John P. Wilson, Charles Ronkon, Edward Burkhardt, Stanley Hoang

**Affiliations:** Department of Neurosurgery, Louisiana State University Health Sciences, Shreveport, Louisiana

**Keywords:** lumbar spondylolisthesis, prone lateral transpsoas interbody fusion, Mazor surgical robot, single-position surgery

## Abstract

Lumbar spondylolisthesis can lead to symptoms of mechanical back pain and radiculopathy. Surgical treatment aims to address dynamic instability and foraminal stenoses. While various alternatives exist to treat this clinical presentation, the single-position prone lateral interbody fusion has recently emerged as a popular technique. This operative video illustrates the treatment of a grade 1 L3–4 spondylolisthesis with a single-position prone lateral transpsoas interbody fusion with percutaneous instrumentation using the Mazor X Stealth robotic system. Detailed step-by-step techniques with operative pearls are provided to help surgeons incorporate the nuances of robotic navigation to the single-position prone lateral procedure.

The video can be found here: https://stream.cadmore.media/r10.3171/2025.4.FOCVID24148

## Transcript

This presentation will demonstrate single-position prone lateral transpsoas interbody fusion with the Mazor X Stealth surgical robotic guidance system.

### 0:28 Clinical Presentation.

We will begin by presenting the patient, who is a 60-year-old male with mechanical back pain and right-sided radiculopathy. His symptoms had been worsening over the last several months and persisted despite physical therapy and epidural steroid injections. On physical exam, there was pain and numbness radiating to his right thigh and foot. He maintained full strength and normal reflexes.

### 0:45 Neuroimaging Findings.

X-ray of the lumbar spine showed a grade 1 L3–4 spondylolisthesis.^1^ MRI of the lumbar spine revealed right-sided L3–4 foraminal stenosis. A scrolling video of the axial T2 and sagittal T2 shows the lateral recess stenosis. Lastly, a CT scan of the lumbar spine showed a collapsed disc space at L3–4.

### 1:04 Rationale for the Procedure.

The surgical plan was for a single-position prone lateral transpsoas interbody fusion at L3–4 with posterior percutaneous fixation using the Mazor surgical robot. The procedure aimed to restore disc space height, reduce and stabilize the L3–4 spondylolisthesis, and achieve interbody fusion.^2^

### 1:19 Alternatives.

Alternatives for this procedure include other lateral and posterior approaches shown here.

### 1:24 Potential Benefits.

Single-position surgery with the Mazor robot offers many benefits, including a more efficient workflow, increased accuracy in placing pedicle screws,^3^ and safer placement of interbody cages with the integrated navigation system. Lateral interbody fusion with the patient in the prone position is more familiar to surgeons and facilitates easier pedicle screw placement and posterior decompression. Additionally, it improves lordosis and sagittal plane correction and provides a safer operative corridor as the lumbar plexus migrates more posteriorly. Furthermore, it is easy to convert to a TLIF without repositioning the patient if lateral transpsoas access is found to be difficult.

### 1:56 Risks of the Procedure.

Challenges to performing the procedure include the associated learning curve with using surgical robot technology^4^ and adapting to single-position prone techniques, which can be associated with inferior drift due to gravity when performing disc preparation. There is also the potential for inaccurate navigation registration. However, this can be mitigated by placing the pedicle screws before the interbody cage. Additional risks include injury to the lumbar plexus or vascular structures, ALL rupture, cage subsidence, and difficulty with clearing the iliac crest or ribs have been described.

### 2:27 Positioning.

The patient was positioned prone on the Jackson Spine Table, with the lateral approach to be performed from the left side. The ipsilateral hip pad was moved more caudally, with the contralateral hip pad on the right side moved more toward the cephalad direction to allow for countertraction when performing disc space preparation and interbody placement. Lastly, the robotic arm was mounted on the right side of the table.

### 2:46 Key Surgical Steps.

For posterior percutaneous instrumentation, the critical steps included obtaining a preoperative CT scan on the morning of surgery to perform a CT to fluoroscopy registration, placement of percutaneous instrumentation, and intraoperative fluoroscopic confirmation. Regarding the lateral interbody fusion, the critical steps included proper registration of navigated instruments, followed by skin incision, dissection through abdominal muscles, identification of the psoas, and docking of dilators into the disc space with neuromonitoring to avoid the lumbar plexus.

### 3:12 Positioning the Patient.

Now, we will present the operative footage. Beginning with the table setup, one can notice that the Jackson Spine Table was set to the highest rung. All pressure points were adequately padded, and the patient was secured to the operative table by taping across the chest and greater trochanters. Additionally, taping was performed to retract excess tissue.

### 3:29 Operative Room Setup and Surgical Planning.

Regarding the operative room setup, the tracking camera was situated at the head of the bed to allow adequate viewing of the robot effector arm and navigated instruments.^5^ The effector arm of the robot was then mounted on the right side at the foot of the bed before skin preparation. After loading the preoperative CT scan into the Mazor software, screw trajectories and dimensions were planned at both levels and the interbody cage was planned to be placed in the ideal position in the disc space.^6^

### 3:51 Robot Registration.

After prepping and draping the patient, the Schanz pin was placed into the right posterior superior iliac spine (PSIS), and a percutaneous pin was placed into the left PSIS. The Schanz pin allowed the robotic arm to be mounted directly to the patient, while the percutaneous pin allowed a different reference frame to be attached for the lateral approach. The bone mount bridge was then connected to the Schanz pin to allow the robotic arm to be linked to the patient’s anatomy to begin the registration process. We then placed blue towels over the surgical area and pointed the operating lights away to perform the 3D scan to define the working surgical area. After removing the blue towels, we attached the star marker to the effector arm and performed a snapshot to merge the navigation system to the robotic arm. We then defined the region of interest by placing the navigation probe at the cranial and caudal boundaries of the incision. Next, we obtained AP and oblique fluoroscopy images for registration. We began by attaching the 3D marker to the effector arm of the robot. The effector arm was then moved into an AP position such that all levels to be operated on were included in the fluoroscopy image, and the entire 3D marker pattern was also visible. After obtaining the AP image, the same step was repeated for the oblique image. After the AP and oblique images were obtained, the software then performed segmentation, labeling, and registration of all levels of interest. Following registration, we verified the merging process before proceeding with the operation.

### 5:08 Posterior Percutaneous Instrumentation.

We then began placing percutaneous instrumentation. The effector arm was moved to the planned trajectory at the desired level of interest. A linear incision was made after a marking pen was used to dot the skin’s entry points. Dissection was taken through the fascia with electrocautery. A specialized No. 11 blade scalpel was placed through the effector arm to make a stab incision inferiorly and superiorly through the fascia, ensuring that bone was felt at the tip of the blade. Next, a navigated dilator and outer cannula were inserted through the effector arm until bone was palpated. The dilator was then removed, leaving the outer cannula in place. An electric navigated drill was placed down the outer cannula into the pedicle to a 30-mm positive stop. The navigated drill was removed, followed by the withdrawal of the outer cannula. A navigated screw was then placed into the effector arm along the planned trajectory into the pedicle to a predetermined depth. No tapping was performed since we used self-tapping screws for this procedure. The effector arm was then moved to the subsequent screw trajectory, and the process was repeated for all 4 screws.

### 6:03 Lateral Approach.

Following the placement of all screws, we turned our attention to the lateral approach. We began registration for the lateral instruments by attaching a reference frame to the left PSIS pin and removing the reference frame attached to the robot. We marked the position of the reference frame to detect any unintentional movement. We then attached the star marker to the effector arm and performed a snapshot to register the navigation system. We then began exposure for the lateral approach. Using navigation, we aligned the skin entry point to the trajectory to the disc space and delineated a 4-cm vertical incision. Dissection proceeded through the skin and subcutaneous tissue. The navigation probe was used throughout the dissection to maintain proper trajectory and avoid inferior drift. Using two Gelpi retractors in the vertical and horizontal positions, we deepened them further for tissue retraction while approaching the retroperitoneal space. The Kelly clamp was then used to spread the muscle fibers of the external oblique, internal oblique, and transversalis muscle and fascia. Once the retroperitoneal space was reached, blunt finger dissection was used to move the retroperitoneal contents anteriorly prior to accessing the disc space. After accessing the disc space, we used the Kittner and Penfield 4 to move the remnant of the psoas muscle anteriorly to delineate the disc space.

### 7:10 Accessing the Disc Space.

Once a safe retroperitoneal corridor was established, the navigation probe was used to approach the disc space. Since this was a collapsed disc space, gentle tapping of the probe with the handle of the mallet allowed the navigation images to remain visible while breaking through the osteophytes. After the disc space was entered, the navigation array was removed, and the probe was placed into the disc space using a mallet. We then placed the first dilator through the navigation probe. We used directional EMG posteriorly and anteriorly to confirm clear proximity of the lumbar plexus. Sequential dilators were then docked in a stepwise manner to place the lateral retractor instrument. Simultaneously, the assisting surgeon can attach the arm to the operating room table to secure the lateral retractor. The lateral retractor was placed, and the sequential dilators were removed. The lighting system was inserted while the assisting surgeon secured the arm. The lateral retractors were then opened and toed out for better access of instruments. We then removed the probe from the disc space, performed the annulotomy with an 11-blade, and removed the remaining disc material using a pituitary. Now, we registered the small 10-mm Cobb instrument with the navigation system and passed it through the disc space along the entirety of both endplates, making sure to break the contralateral annulus. This was repeated using the larger 20-mm Cobb. It is important that the Cobb passes through and breaks the contralateral annulus to aid in annular release, deformity correction, and indirect decompression. To further access the disc space, we used a 4-mm and 6-mm distractor to assist in widening the disc space. To clear the disc space further, a straight curette was used to scrape along the superior endplate followed by turning the curette 180° to go along the inferior endplate.

### 8:43 Interbody Trial and Cage Placement.

After the disc space has been sequentially distracted with trials until adequate disc space height has been obtained and adequate foraminal size has been restored, we selected a trial with appropriate width, length, and lordosis angle based on preoperative CT measurement. In this case, an 8-mm-height trial was used prior to cage insertion. Following removal of the trial, the cleared disc space could be seen in this image prior to interbody placement. We then placed a cage with a height of 10 mm and 6° of lordosis using navigation. The still image here shows the cage within the disc space.

### 9:20 Confirmation of Cage Placement.

AP and lateral fluoroscopy were then taken to confirm optimal cage placement. Before closing the lateral incision, we obtained an O-arm spin to confirm proper placement of the interbody cage and screws. The lateral incision was closed while the posterior surgeon finished the remaining steps by placing the percutaneous rods and final tightening of the screws.

### 9:37 Lumbar Spondylolisthesis.

In summary, this case presented a patient whose symptoms included mechanical back pain and right-sided radiculopathy as a result of lumbar spondylolisthesis. Surgical treatment to correct this pathology should address the associated instability and radiculopathy resulting from nerve root compression. Restoring physiological disc height can improve foraminal stenoses and lead to indirect decompression. Moreover, posterior percutaneous fixation improves the stability of the construct and promotes arthrodesis.

### 10:01 Clinical Outcome.

Intraoperative fluoroscopic images show restoration of the disc space height and reduction of the spondylolisthesis at L3–4. Postop CT confirms proper placement of the interbody cage and pedicle screws. Comparison of preop versus postop CT demonstrates the change in height of the right L3–4 foramen as a result of indirect decompression.

The patient reported significant postoperative improvement in radiculopathy. Additionally, there were no complaints with the transpsoas approach, and the patient reported no psoas weakness or sensory disturbance postoperatively. The patient was discharged home on postop day 2. The patient continued to do well at the 10-month follow-up visit.

## Disclosures

Dr. Hoang reported personal fees from Medtronic to teach prone lateral courses outside the submitted work.

## Author Contributions

Primary surgeon: Hoang. Assistant surgeon: Burkhardt. Editing and drafting the video and abstract: Hoang, Wilson. Critically revising the work: all authors. Reviewed submitted version of the work: all authors. Approved the final version of the work on behalf of all authors: Hoang.

## Supplemental Information

### Patient Informed Consent

The necessary patient informed consent was obtained in this study.
